# Antimicrobial Natural Products from Plant Pathogenic Fungi

**DOI:** 10.3390/molecules28031142

**Published:** 2023-01-23

**Authors:** Melissa M. Cadelis, Steven A. Li, Shara J. van de Pas, Alex Grey, Daniel Mulholland, Bevan S. Weir, Brent R. Copp, Siouxsie Wiles

**Affiliations:** 1 School of Chemical Sciences, University of Auckland, Private Bag 92019, Auckland 1142, New Zealand; 2 Bioluminescent Superbugs Lab, School of Medical Sciences, University of Auckland, Private Bag 92019, Auckland 1142, New Zealand; 3 Manaaki Whenua—Landcare Research, Private Bag 92170, Auckland 1142, New Zealand; 4 Te Pūnaha Matatini Centre of Research Excellence in Complex Systems, Auckland 1142, New Zealand

**Keywords:** antibacterial activity, *Candida albicans*, fungal plant pathogens, isolation, metabolites, mycobacteria, natural product, *Staphylocccus aureus*

## Abstract

Isolates of a variety of fungal plant pathogens (*Alternaria radicina* ICMP 5619, *Cercospora beticola* ICMP 15907, *Dactylonectria macrodidyma* ICMP 16789, *D. torresensis* ICMP 20542, *Ilyonectria europaea* ICMP 16794, and *I. liriodendra* ICMP 16795) were screened for antimicrobial activity against the human pathogenic bacteria *Acinetobacter baumannii*, *Pseudomonas aeruginosa*, *Escherichia coli*, *Mycobacterium abscessus*, and *M. marinum* and were found to have some activity. Investigation of the secondary metabolites of these fungal isolates led to the isolation of ten natural products (**1–10**) of which one was novel, (*E*)-4,7-dihydroxyoct-2-enoic acid (**1**). Structure elucidation of all natural products was achieved by a combination of NMR spectroscopy and mass spectrometry. We also investigated the antimicrobial activity of a number of the isolated natural products. While we did not find (*E*)-4,7-dihydroxyoct-2-enoic acid (**1**) to have any activity against the bacteria and fungi in our assays, we did find that cercosporin (**7**) exhibited potent activity against Methicillin resistant *Staphylococcus aureus* (MRSA), dehydro-curvularin (**6**) and radicicol (**10**) exhibited antimycobacterial activity against *M. marinum*, and brefeldin A (**8**) and radicicol (**10**) exhibited antifungal activity against *Candida albicans*. Investigation of the cytotoxicity and haemolytic activities of these natural products (**6–8** and **10**) found that only one of the four active compounds, radicicol (**10**), was non-cytotoxic and non-haemolytic.

## 1. Introduction

Plant pathogenic fungi have evolved to invade and kill living plant tissue to derive nutrition and facilitate their reproduction. Entry of fungi into living plant tissue can be though natural openings such as leaf stomata [1] or using specialised fungal structures such as appressoria to punch holes though protective plant cell walls [2]. Once inside the plant cell, fungal pathogens must suppress or evade plant defensive responses typically though protein–protein interactions comprising fungal effectors and specific matching host plant resistant (R) proteins [3].

The role of secondary metabolites in this process is under explored but they can act as phytotoxins that enhance pathogenicity and virulence [4] and as mycotoxins that suppress endophytic and other competing phytopathogenic fungi [5]. In our ongoing investigation of the secondary metabolites of several necrotrophic plant pathogens from the International Collection of Microorganisms from Plants (ICMP), namely, *Alternaria radicina*, *Cercospora beticola*, *Dactylonectria macrodidyma*, *D. torresensis*, *Ilyonectria europaea,* and *I. liriodendra,* we isolated natural products **1–10**, one of which was novel. Herein, we report the fermentation, isolation, and biological activities of these natural products.

## 2. Results

**Figure 1:**
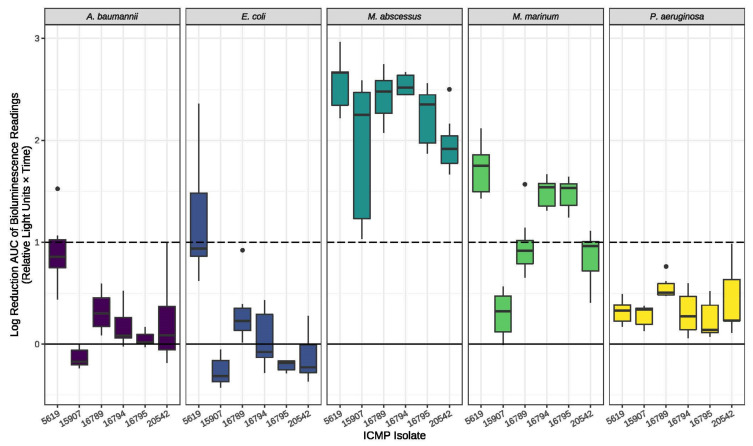
Antibacterial activity of ICMP 5619, 15907, 16789, 16794, 16795, and 20542 against *A. baumannii*, *E. coli*, *M. abscessus*, *M. marinum*, and *P. aeruginosa*. Data are presented as box and whisker plots of the activity scores. The solid line shown at 0 is the median control value while the dotted line at 1 is the activity threshold. Scores above 1 correspond to a >90% reduction in bacterial bioluminescence compared to the corresponding no-fungi control. Similarly, an activity score above 2 corresponds to a >99% reduction.

We screened several plant pathogens from the ICMP collection for antimicrobial activity against bioluminescent derivatives of *Acinetobacter baumannii*, *Pseudomonas aeruginosa*, *Escherichia coli*, *Mycobacterium abscessus*, and *M. marinum*. Antibacterial activity was measured as log reductions in light output compared to no-fungi controls and are presented as activity scores. Scores above 1 correspond to a >90% reduction in bacterial bioluminescence compared to the corresponding no-fungi control. Similarly, an activity score above 2 corresponds to a >99% reduction. ICMPs 5619, 15907, 16789, 16794, 16795, and 20542 were identified as hits against both *M. abscessus* with ICMPs 5619, 16794, and 16795 also exhibiting activity against *M. marinum* (Figure 1). In addition, ICMP 5619 was also found to be active against *E. coli*. Thus, the fungal isolates *Alternaria radicina* ICMP 5619, *Cercospora beticola* ICMP 15907, *Dactylonectria macrodidyma* ICMP 16789, *D. torresensis* ICMP 20542, *Ilyonectria europaea* ICMP 16794, and *I. liriodendra* ICMP 16795 were selected for further investigation.

**Figure 2:**
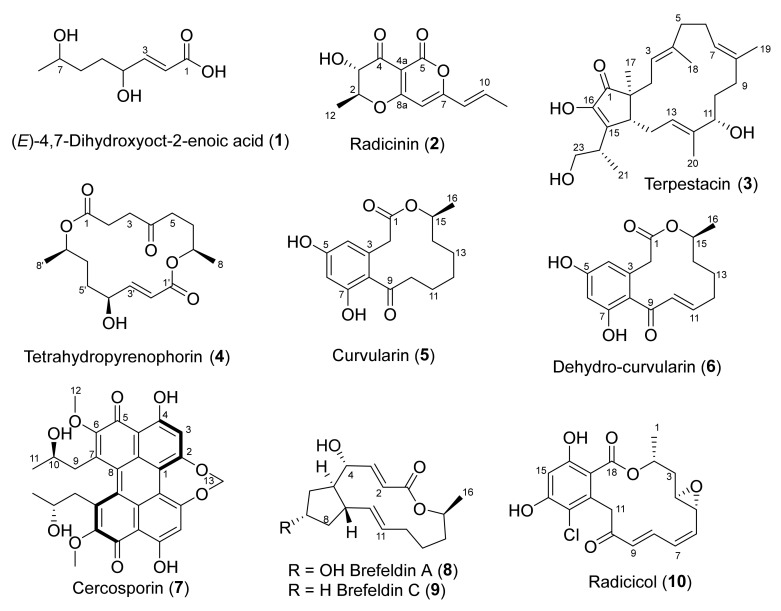
Structures of the isolated natural products **1–10**.

ICMP 5619, 15907, 16789, 16794, 16795, and 20542 were incubated and grown on potato dextrose agar (PDA) plates and extracted with a combination of methanol and dichloromethane to afford crude extracts. The crude extracts were subjected to extensive chromatographic methods for purification including C_8_ reversed-phase column chromatography (H_2_O/MeOH), Sephadex LH-20 (MeOH/5% CH_2_Cl_2_) and Diol-bonded silica gel (hexane/EtOAc) chromatography to afford compounds **1–10** (Figure 2 and Table 1). 

**Figure 3:**
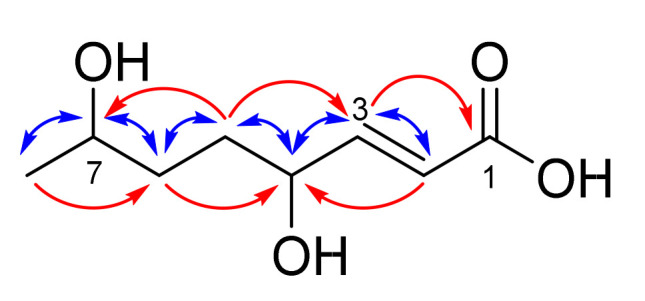
Selected COSY correlations (blue) and HMBC correlations (red) for **1**.

High resolution ESIMS of **1**, isolated from *Alternaria radicina* ICMP 5619, showed the presence of a sodiated adduct at *m*/*z* 197.0784 corresponding to a molecular formula of C_8_H_14_O_4_. Analysis of the proton NMR spectrum of **1** in MeOD (Figure S1) showed the presence of two olefinic protons (δ_H_ 6.87 (dd, *J* = 15.6, 6.3 Hz, H-3) and 5.90 (dd, *J* = 15.6, 1.4 Hz, H-2)), two oxygenated methines (δ_H_ 5.04 (m, H-7) and 4.12 (m, H-4)), two sets of diastereotopic methylenes (δ_H_ 1.80 (m, H-5a), 1.71 (m, H-5b, H-6a), and 1.60 (m, H-6b)) and a methyl group (δ_H_ 1.25 (d, *J* = 6.5 Hz, H_3_-8). The carbon NMR spectrum (Figure S2) showed eight carbon signals including a carbonyl carbon (δ_C_ 167.3 (C-1)), two olefinic carbons (δ_C_ 151.0 (C-3) and 122.7 (C-2)), two oxygenated methines (δ_C_ 71.4 (C-4/C-7) and 71.3 (C-4/C-7)), two methylenes (δ_C_ 31.8 (C-5) and 29.5 (C-6)), and a methyl carbon (δ_C_ 18.9 (C-8)). COSY cross correlations (Figure S3) established the connectivity order across the oct-2-enyl chain with the two olefinic protons being attributed to an *E*-alkene due to the relatively large *J*_HH_ coupling constant of 15.6 Hz. HBMC correlations (Figure S5) observed between H_3_-8 and C-6, H_2_-5 and C-7, H-3 and both C-5 and C-1 and between H-2 and C-4 provided further evidence of connectivity (Figure 3). Thus, the structure of compound **1** was established as (*E*)-4,7-dihydroxyoct-2-enoic acid. The stereochemistry at C-4 and C-7 for **1** could not be assigned as **1** was optically inactive and showed no absorption curves in the electronic circular dichroism experiment.

Structure elucidation of compounds **1–10** was achieved by a combination of NMR spectroscopy and mass spectrometry, which was in agreement with the literature [6,7,8,9,10,11,12]. In addition to **1**, *Alternaria radicina* ICMP 5619 also afforded compounds **2–6**, which were identified as radicinin (**2**) [13], terpestacin (**3**) [14], tetrahydropyrenophorin (**4**) [15], curvularin (**5**) [16] and dehydro-curvularin (**6**) [17]. Compound **7**, isolated from *Cercospora beticola* ICMP 15907 was identified as cercosporin (**7**) [18] while compound **8**, isolated from both *D. macrodidyma* ICMP 16789 and *D. torresensis* ICMP 20542, was identified as brefeldin A (**8**) [19] with the latter also producing brefeldin C (**9**) [20]. Both *I. europaea* ICMP 16794 and *I. liriodendra* ICMP 16795 afforded compound **10**, which was identified as radicicol (**10**) [21].

All compounds except for **9**, due to insufficient sample, were evaluated for their antimicrobial activity against a panel of bacterial (*A. baumannii*, *E. coli*, *Klebsiella pneumoniae*, Methicillin-resistant *Staphylococcus aureus* (MRSA), and *P. aeruginosa*) and fungal (*Candida albicans* and *Cryptococcus neoformans*) pathogens (Table 1). Of all the compounds tested, cercosporin (**7**) was the most active against the bacterial strains with a MIC of ≤0.47 µM against MRSA. Intriguingly, the activity of ICMP 5619 we observed against *E coli* during initial screening was lost after purification, not an uncommon occurrence. Of note was the moderate antifungal activity exhibited by brefeldin A (**8**) and radicicol (**10**) against *C. albicans* with minimum inhibitory concentrations (MICs) of 57 and 44 µM, respectively. Neither of these compounds exhibited significant activity against any of the bacterial strains.

All compounds were also evaluated for their cytotoxicity against human embryonic kidney cells (HEK-293) and any haemolytic activity against human red blood cells. Compounds **6–8** were found to be cytotoxic with 50% cytotoxic concentrations (CC_50_) of 1.27, 25.33, and 0.89 µM, respectively. Compound **10** was non-cytotoxic and non-haemolytic.

**Figure 4:**
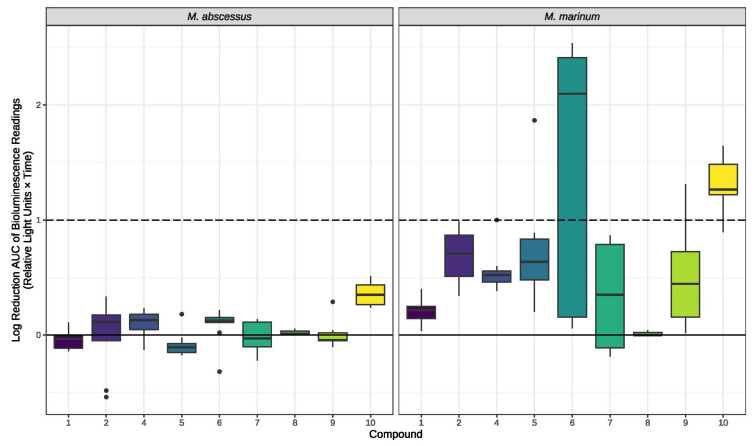
Antimycobacterial activity of compounds **1**, **2**, and **4–10** against *M. abscessus* BSG301 and *M. marinum* BSG101. Data are presented as box and whisker plots of activity scores. The solid line shown at 0 is the median control value while the dotted line at 1 is the activity threshold. Scores above 1 correspond to a >90% reduction in bacterial bioluminescence compared to the corresponding no-fungi control. Similarly, an activity score above 2 corresponds to a >99% reduction.

The antimycobacterial activity of compounds **1**, **2**, and **4–10** was also investigated using bioluminescent strains of *M. abscessus* and *M. marinum* (Figure 4). Compound **3** was not investigated due to the lack of sample. In contrast to the observed antimycobacterial activity observed in the initial screening, none of the tested compounds showed activity against *M. abscessus* or *M. marinum* except for dehydro-curvularin (**6**) and radicicol (**10**), which showed activity against *M. marinum* (MIC of 32 and 64 μg/mL, respectively).

## 3. Discussion

Secondary metabolites from the fungal plant pathogens *Alternaria radicina*, *Cercospora beticola*, *D. macrodidyma*, *D. torresensis*, *I. europaea*, and *I. liriodendra* have been reported to exhibit a variety of bioactivities including antimicrobial, anti-proliferative, antimalarial, and phytotoxic activities and as inhibitors of acetylcholinesterase [22,23,24,25,26,27,28,29,30,31,32,33,34].

In this study, we isolated ten natural products from these fungi including the novel compound (*E*)-4,7-dihydroxyoct-2-enoic acid (**1**), and the known compounds radicinin (**2**) [13], terpestacin (**3**) [14], tetrahydropyrenophorin (**4**) [15], curvularin (**5**) [16], dehydro-curvularin (**6**) [17], cercosporin (**7**) [18], brefeldin A (**8**) [19], brefeldin C (**9**) [20] and radicicol (**10**) [21].

The antimicrobial activities of radicinin (**2**), tetrahydropyrenophorin (**4**), curvularin (**5**), dehydro-curvularin (**6**), brefeldin A (**8**), and radicicol (**10**) have previously been reported (Table 2). Radicinin (**2**) has been shown to exhibit moderate antifungal activity against *Elymus repens* and *Mycotypha microspora* [35] while tetrahydropyrenophorin (**4**) has been shown to exhibit moderate antibacterial activity against *E. coli* and *Bacillus megaterium* as well as antifungal activity against *Microbotryum violaceum* [36]. Interestingly, we could not replicate this anti-*E. coli* activity in the present study. This could be because of the differences in the methods used to measure antibacterial activity where Zhang et al. used an agar diffusion assay with an unspecified bacterial inoculum sprayed onto the agar plates [36] while we performed our assays in a liquid medium. It is well-known that factors such as inoculum size [37] can impact on the results of antimicrobial activity testing, so this may be the reason for the discrepancy. Betina and Mičeková [38] previously investigated the antimicrobial activities of **5**, **8**, and **10** against *E. coli*, *Bacillus subtilis*, *C. albicans*, *Saccharomyces cerevisiae*, and *Botrytis cinerea* in disk diffusion assays and found that none of the compounds were active against *E. coli* and only **10** was active against *B. subtilis*. Our findings agree with this. In addition, **8** has also been reported to exhibit activity against *Aspergillus fumigatus* and *Microsporum gypseum* while **10** has been reported to have activity against *Aspergillus flavus* [38,39,40]. Investigation of the antimicrobial activities of **5** and **6** against *B. subtilis*, *S. aureus*, *S. cerevisiae, Sclerotinia sclerotiorum,* and *Mycobacteria tuberculosis* showed that both compounds were inactive against all strains with the exception of **6**, which was active against *M. tuberculosis* (MIC 40 μM) [9,26].

We tested the novel compound (*E*)-4,7-dihydroxyoct-2-enoic acid (**1**) from *Alternaria radicina* ICMP 5619 and while we found it to have no cytotoxic or haemolytic activity, it also possessed no antimicrobial activity against the bacterial and fungal strains we used. Of the other known natural products isolated, we found that compound **7** exhibited potent activity against MRSA and **6** and **10** had activity against *M. marinum.* This last finding is in keeping with the findings of Souza et al., who determined that compound **6** was active against another *Mycobacterium* species, *M. tuberculosis* [26]. Meanwhile, compounds **8** and **10** exhibited moderate antifungal activity against *C. albicans.* Unfortunately, compounds **6–8** were found to be cytotoxic but not haemolytic, while **10** exhibited no cytotoxicity or haemolytic activity.

**Table 2:** Summary of natural products **1–10** isolated from fungal pathogens.

Fungus	ICMP	Compounds Isolated	Bioactivity	References
*Alternaria radicina*	5619	(*E*)-4,7-dihydroxyoct-2-enoic acid (**1**)	None	
		Radicinin (**2**)	Phytotoxic Antifungal Anti-proliferative Acetylcholinesterase inhibitor	[27,30,32,33,35]
		Terpestacin (**3**)	Anti-proliferative	[41]
		Tetrahydropyrenophorin (**4**)	Antibacterial Antifungal Algicidal	[36]
		Curvularin (**5**)	Phytotoxic	[9,23,26,38,42]
		Dehydrocurvularin (**6**)	Antifungal Phytotoxic Antimycobacterial	[9,23,26,42]
*Cercospora beticola*	15907	Cercosporin (**7**)	Photo-activated plant toxin	[22,43]
*Dactylonectria macrodidyma*	16789	Brefeldin A (**8**) (also known as Decumbin, Cyanein, Ascotoxin, Nectrolide, Synergisidin)	Anti-proliferative Antifungal	[19,38,44,45,46]
		Brefeldin C (**9**)		[20]
*Dactylonectria torresensis*	20542	Brefeldin A (**8**)	Anti-proliferative	[19,38,44,45,46]
*Ilyonectria* *europaea*	16794	Radicicol (**10**) (also known as Monorden)	HSP90 inhibitor Antifungal Antimalarial	[24,25,34,39,47]
*Ilyonectria* *liriodendra*	16795	Radicicol (**10**)	HSP90 inhibitor Antifungal Antimalarial	[24,25,34,39,47]

## 4. Materials and Methods

### 4.1. General Experimental Procedures

Mass spectra were acquired on a Bruker micrOTOF Q II spectrometer. Specific rotations were recorded on an Autopol IV polarimeter using a 1 dm cell (concentration units of g/100 mL). Melting points were recorded on an electrothermal melting point apparatus and were uncorrected. Electronic circular dichroism readings were obtained with a Chirascan circular dichroism spectrometer using a 1 mm cuvette (concentration units of molL^−1^). ^1^H and ^13^C NMR spectra were recorded at 298 K on a Bruker AVANCE 400 spectrometer at 400 and 100 MHz, respectively, using standard pulse sequences. Proto-deutero solvent signals were used as internal references (CD_3_OD: δ_H_ 3.31, δ_C_ 49.0; (CD_3_)_2_CO: δ_H_ 2.04, δ_C_ 29.8; CDCl_3_: δ_H_ 0.00 (TMS), δ_C_ 77.16). For ^1^H NMR, the data are quoted as position (δ), relative integral, multiplicity (s = singlet, d = doublet, t = triplet, q = quartet, p = pentet, m = multiplet, dd = doublet of doublets, ddd = doublet of doublets of doublets, dt = doublet of triplets, dq, = doublet of quartets, br = broad), coupling constant (*J*, Hz), and assignment to the atom. The ^13^C NMR data are quoted as position (δ) and assignment to the atom. Flash column chromatography was carried out using Kieselgel silica gel (40–63 μm) or Merck diol bonded silica (40–63 μm), C_8_ (Merck) reversed-phase (40–63 μm) solid support. Gel filtration flash chromatography was carried out on Sephadex LH–20 (Pharmacia). Thin layer chromatography was conducted on DC–plastikfolien Kieselgel 60 F254 plates. All solvents used were of analytical grade or better and/or purified according to the standard procedures.

### 4.2. Fungal Material

Fungal isolates of *Alternaria radicina*, *Cercospora beticola*, *Dactylonectria macrodidyma*, *D. torresensis*, *Ilyonectria europaea*, and *I. liriodendra* were acquired from Manaaki Whenua—Landcare Research’s International Collection of Microorganisms from Plants (ICMP).

#### 4.2.1. ICMP 5619—*Alternaria radicina*

*A. radicina* is a pathogen of carrot (*Daucus carota*) causing the disease ‘black rot’. It is a globally common fungus associated with carrot production [48]. Culture ICMP 5619 was isolated in February 1969 from a diseased carrot in Ohakune, the major carrot growing region of New Zealand. The identification of this culture is supported by GenBank sequence MW862781 [49].

#### 4.2.2. ICMP 15907—*Cercospora beticola*

*C. beticola* is described as a leaf spot pathogen of sugar beet (*Beta vulgaris*), however, it has been recorded on eight different hosts in New Zealand [50]. Culture ICMP 15907 was isolated in April 2005 from lupin (*Lupinus polyphyllus*) in Auckland, New Zealand. The identification of this culture is supported by GenBank sequences OP390248, OP382645, OP382665, OP382685, and OP382702 [51].

#### 4.2.3. ICMP 16789—*Dactylonectria macrodidyma*

*D. macrodidyma* is a plant pathogen affecting the roots of grape vines causing the disease ‘black foot’, and it is occasionally isolated from other plants. It is found to be associated with grapevines in Australia, Europe, New Zealand, South Africa, and the USA [52]. Culture ICMP 16789 was isolated in May 2005 from a dead grapevine in Gisborne, New Zealand. The identification of this culture is supported by GenBank sequence MH553533 [53].

#### 4.2.4. ICMP 20542—*Dactylonectria torresensis*

*D. torresensis* is a plant root pathogen causing disease on a wide variety of hosts worldwide [54]. Culture ICMP 20542 was isolated in March 2005 from a grapevine root in Waipara, New Zealand. The identification of this culture is supported by GenBank sequences MW862792 and MZ393468 [55].

#### 4.2.5. ICMP 16794—*Ilyonectria europaea*

*I. europaea* is a plant root pathogen. It is widely distributed in Europe on several different plant species. In New Zealand, it has been found on grapevine and apple. Culture ICMP 16794 was isolated in March 2005 from a grapevine root in Waipara, New Zealand. The identification of this culture is supported by GenBank sequences MH497571 and MH553543 [56].

#### 4.2.6. ICMP 16795—*Ilyonectria liriodendri*

*I. liriodendri* is a significant root disease of grapevines both in New Zealand and worldwide [57]. Culture ICMP 16795 was isolated in March 2005 from a grapevine root in Waipara, New Zealand. The identification of this culture is supported by GenBank sequences MW862788 and MZ393467 [58].

### 4.3. Fermentation, Extraction and Isolation

#### 4.3.1. ICMP 5619—*Alternaria radicina*

Forty PDA plates were inoculated with ICMP 5619 and incubated at room temperature for 3 weeks. Fully grown fungal plates were freeze-dried (20.42 g, dry weight) and extracted with MeOH (2 × 500 mL) for 4 h followed by CH_2_Cl_2_ (500 mL) overnight. Combined organic extracts were concentrated under reduced pressure to afford a brown oil (2.05 g). The crude product was subjected to purification by C_8_ reversed-phase column chromatography eluted with a gradient of H_2_O/MeOH to afford five fractions (F1–F5). Purification of Fraction F3 by silica gel column chromatography, eluted with *n*-hexane/EtOAc (1:1), afforded four fractions (D1–D4). Fraction D3 was triturated with water to afford radicinin (**2**) as a white solid (2.50 mg), while the eluent afforded (*E*)-4,7-dihydroxyoct-2-enoic acid (**1**) as a yellow oil (2.0 mg). Fraction F4 was subjected to purification by silica gel column chromatography and eluted with CH_2_Cl_2_/MeOH (gradient) to afford our fractions (A1–A4). Purification of A1 by silica gel column chromatography, eluted with *n*-hexane/EtOAc (1:1), afforded four fractions (B1–B4). Fraction B1 afforded dehydro-curvularin (**6**) as a yellow solid (50.2 mg) while B2 afforded curvularin (**5**) as a white solid (35.6 mg). Fraction B3 was subjected to Sephadex LH-20, eluted with MeOH to afford three fractions (C1–C3). Subsequent purification of C1 by diol-bonded silica gel column chromatography, eluted with EtOAc/PetEther (gradient), afforded tetrahydropyrenophorin (**4**) (5.5 mg) and terpestacin (**3**) (1.2 mg).

##### (*E*)-4,7-Dihydroxyoct-2-enoic acid (**1**)

[α]_D_^19^ = −5.9 (*c* = 0.27, MeOH); ^1^H NMR (CD_3_OD, 400 MHz) δ 6.87 (1H, dd, *J* = 15.6, 6.3 Hz, H-3), 5.90 (1H, dd, *J* = 15.6, 1.4 Hz, H-2), 5.04 (1H, m, H-7), 4.12 (1H, m, H-4), 1.80 (1H, m, H-5a), 1.71 (2H, m, H-5b, H-6a), 1.60 (1H, m, H-6b), 1.25 (3H, d, *J* = 6.5 Hz, H_3_-8); ^13^C NMR (CD_3_OD, 100 MHz) δ 167.3 (C-1), 151.0 (C-3), 122.7 (C-2), 71.4 (C-4/C-7), 71.3 (C-4/C-7), 31.8 (C-5), 29.5 (C-6), 18.9 (C-8); (+)-HRESIMS *m*/*z* 197.0784 [M + Na]^+^ (calcd for C_8_H_14_NaO_4_, 197.0784).

##### Radicinin (**2**)

[α]_D_^20^ = −43.1 (*c* = 0.35, CHCl_3_/EtOH 2:1) [lit [α]_D_^22^ = −125 (*c* = 1.26, CHCl_3_/EtOH 2:1) [59]]; ^1^H NMR (CDCl_3_, 400 MHz) δ 6.98 (1H, dq, *J* = 15.5, 7.0 Hz, H-10), 6.04 (1H, dq, *J* = 15.5, 1.5 Hz, H-9), 5.85 (1H, s, H-8), 4.36 (1H, dq, *J* = 12.5, 6.1 Hz, H-2), 3.99 (1H, d, *J* = 12.5 Hz, H-3), 3.84 (1H, br s, OH), 1.97 (1H, dd, *J* = 7.0, 1.5 Hz, H-11), 1.66 (1H, d, *J* = 6.1 Hz, H-12); (+)-HRESIMS *m*/*z* 259.0590 [M + Na]^+^ (calcd for C_12_H_12_NaO_5_, 259.0582).

##### Terpestacin (**3**)

[α]_D_^22^ = −7.3 (*c* = 0.21, MeOH) [lit [α]_D_ = −18 (*c* = 0.1, MeOH) [60]]; ^1^H NMR (CDCl_3_, 400 MHz) δ 5.41–5.39 (1H, m, H-13), 5.26–5.23 (1H, m, H-3), 5.15–5.13 (1H, m, H-7), 4.06 (1H, dd, *J* = 9.8, 3.8 Hz, H-11), 3.89 (1H, dd, *J* = 10.4, 7.3 Hz, H_2_-23a), 3.82 (1H, dd, *J* = 10.4, 7.3 Hz, H_2_-23b), 2.71 (1H, dd, *J* = 11.5, 2.5 Hz, H-14a), 2.68–2.65 (1H, m, H-22), 2.47–2.42 (1H, m, H_2_-14a), 2.39 (1H, dd, *J* = 13.8, 10.5 Hz, H_2_-2a), 2.29–2.24 (2H, m, H_2_-5a, H_2_-6a), 2.23–2.19 (1H, m, H_2_-9a), 2.13–2.07 (1H, m, H_2_-6b), 2.05–2.00 (2H, m, H_2_-5b, H_2_-9b), 1.98–1.91 (1H, m, H_2_-14b), 1.80–1.73 (2H, m, H_2_-10), 1.72–1.67 (1H, m, H_2_-2b), 1.64 (6H, br s, H_3_-18, H_3_-19), 1.57 (3H, s, H_3_-20), 1.29 (3H, d, *J* = 7.3 Hz, H_3_-21), 1.00 (3H, s, H_3_-17); HRESIMS *m/z* 425.2663 [M + Na]^+^ (calcd for C_25_H_38_NaO_4_, 425.2662).

##### Tetrahydropyrenophorin (**4**)

[α]_D_^22^ = −11.0 (*c* = 0.10, CHCl_3_) [lit [α]_D_^20^ = −11.0 (*c* = 0.21, CHCl_3_) [36]]; ^1^H NMR (CDCl_3_, 400 MHz) δ 6.77 (1H, dd, *J* = 16.0, 6.5 Hz, H-3′), 5.89 (1H, dd, *J* = 16.0, 1.0 Hz, H-2′), 4.94–4.87 (2H, m, H-7, H-7′), 4.30–4.26 (1H, m, H-4′), 2.90–2.83 (1H, m, H-3a), 2.77–2.68 (1H, m, H-2a), 2.63 (1H, dd, *J* = 8.3, 3.2 Hz, H-5a), 2.52 (1H, dd, *J* = 8.6, 3.2 Hz, H-5b), 2.51–2.47 (1H, m, H-3b), 2.41–2.38 (1H, m, H-2b), 2.17–2.10 (1H, m, H-6), 1.83–1.78 (1H, m, H-5′a), 1.78–1.75 (1H, m, H-5′b), 1.70–1.66 (1H, m, H-6′a), 1.61–1.57 (1H, m, H-6′b), 1.28 (3H, d, *J* = 5.8 Hz, H_3_-8), 1.22 (3H, d, *J* = 6.3 Hz, H_3_-8′); ^13^C NMR (CDCl_3_, 100 MHz) δ 207.8 (C-4), 172.4 (C-1), 165.9 (C-1′), 149.3 (C-3′), 122.7 (C-2′), 71.9 (C-7), 71.3 (C-4′), 70.3 (C-7′), 39.3 (C-5), 37.5 (C-3), 30.1 (C-5′), 29.9 (C-6′), 28.9 (C-6), 28.7 (C-2), 20.5 (C-8), 18.9 (C-8′); (+)-HRESIMS *m*/*z* 335.1473 [M + Na]^+^ (calcd for C_16_H_24_NaO_6_, 335.1471).

##### Curvularin (**5**)

[α]_D_^22^ = −30 (*c* = 0.1, MeOH) [lit [α]_D_^20^ = −44.9 (*c* = 1.0, MeOH) [61]]; ^1^H NMR ((CD_3_)_2_CO, 400 MHz) δ 6.39 (1H, d, *J* = 2.4 Hz, H-4), 6.34 (1H, d, *J* = 2.4 Hz, H-6), 4.92 (1H, m, H-15), 3.78 (1H, d, *J* = 16.0 Hz, H-2a), 3.70 (1H, d, *J* = 16.0 Hz, H-2b), 3.11 (1H, ddd, *J* = 15.5, 8.4, 2.9 Hz, H-10a), 2.77 (1H, ddd, *J* = 15.5, 8.4, 2.9 Hz, H-10b), 1.74 (1H, m, H-11a), 1.59 (2H, m, H-14a), 1.51 (1H, m, H-11b), 1.46 (1H, m, H-13a), 1.43 (1H, m, H-14b), 1.41 (1H, m, H-12a), 1.31 (1H, m, H-13b), 1.27 (1H, m, H-12b), 1.11 (3H, d, *J* = 6.2 Hz, H_3_-16); ^13^C NMR ((CD_3_)_2_CO, 100 MHz) δ 206.7 (C-9), 171.0 (C-1), 160.2 (C-5), 158.2 (C-7), 136.9 (C-3), 121.6 (C-8), 112.2 (C-4), 102.5 (C-6), 72.6 (C-15), 44.0 (C-10), 39.7 (C-2), 33.0 (C-14), 27.6 (C-12), 24.6 (C-13), 23.5 (C-11), 20.6 (C-16); (+)-HRESIMS *m*/*z* 315.1203 [M + Na]^+^ (calcd for C_16_H_20_NaO_5_, 315.1203).

##### Dehydro-curvularin (**6**)

[α]_D_^22^ = −48 (*c* = 0.1, MeOH) [lit [α]_D_^20^ = −64.9 (*c* = 1.0, MeOH) [61]]; ^1^H NMR ((CD_3_)_2_CO, 400 MHz) δ 6.78 (1H, d, *J* = 15.5 Hz, H-10), 6.57 (1H, ddd, *J* = 15.5, 8.9, 4.7 Hz, H-11), 6.36 (1H, d, *J* = 2.4 Hz, H-4), 6.31 (1H, d, *J* = 2.4 Hz, H-6), 4.74 (1H, m, H-15), 4.08 (1H, d, *J* = 18.1 Hz, H-2a), 3.61 (1H, d, *J* = 18.1 Hz, H-2b), 2.42 (1H, m, H-12a), 2.35 (1H, m, H-12b), 2.00 (1H, m, H-13a), 1.86 (1H, m, H-14a), 1.66 (1H, m, H-13b), 1.62 (1H, m, H-14b), 1.20 (3H, d, *J* = 6.4 Hz, H_3_-16); ^13^C NMR ((CD_3_)_2_CO, 100 MHz) δ 197.1 (C-9), 171.8 (C-1), 165.9 (C-7), 163.3 (C-5), 149.5 (C-11), 139.4 (C-3), 132.6 (C-10), 115.5 (C-8), 113.8 (C-4), 102.9 (C-6), 72.8 (C-15), 43.7 (C-2), 34.7 (C-14), 33.1 (C-12), 24.9 (C-13), 20.3 (C-16); (+)-HRESIMS *m*/*z* 313.1048 [M + Na]^+^ (calcd for C_16_H_18_NaO_5_, 313.1046).

#### 4.3.2. ICMP 15907—*Cercospora beticola*

Eight PDA plates were inoculated with ICMP 15907 and incubated at room temperature for 4 weeks. Fully grown fungal plates were freeze-dried (4.62 g, dry weight) and extracted with MeOH (2 × 200 mL) for 4 h followed by CH_2_Cl_2_ (200 mL) overnight. Combined organic extracts were concentrated under reduced pressure to afford a brown oil (0.543 g). The crude product was subjected to purification by C_8_ reversed-phase column chromatography eluted with a gradient of H_2_O/MeOH to afford six fractions (F1–F6). F4 was subjected to purification by diol-bonded silica gel column chromatography, eluted with CH_2_Cl_2_/MeOH (gradient) to afford cercosporin (**7**) as a solid (20.4 mg).

##### Cercosporin (**7**)

[α]_D_^19^ = + 256.5 (*c* = 0.03, CHCl_3_) [lit [α]_D_^20^ = + 470 (*c* = 0.50, CHCl_3_) [18]]; ^1^H NMR (CDCl_3_, 400 MHz) δ 14.82 (2H, s, OH-4), 7.07 (2H, s, H-7), 5.74 (2H, s, H-13), 4.21 (6H, s, H-12), 3.59 (2H, dd, *J* = 12.9, 6.4 Hz, H-10), 3.39 (2H, m, H-9a), 2.90 (2H, dd, *J* = 12.9, 5.9 Hz, H-9b), 0.64 (6H, d, *J* = 6.4 Hz, H_3_-11); ^13^C NMR (CDCl_3_, 100 MHz) δ 181.9 (C-5), 167.6 (C-4), 163.5 (C-2), 152.9 (C-6), 135.4 (C-7), 130.7 (C-8), 128.1 (C-8a), 113.1 (C-1), 109.5 (C-3), 108.4 (C-4a), 92.8 (C-13), 68.2 (C-10), 61.3 (C-12), 42.3 (C-9), 23.5 (C-11); (+)-HRESIMS *m*/*z* 557.1424 [M + Na]^+^ (calcd for C_29_H_26_NaO_10_, 557.1418).

#### 4.3.3. ICMP 16789—*Dactylonectria macrodidyma*

Nineteen PDA plates were inoculated with ICMP 16789 and incubated at room temperature for 4 weeks. Fully grown fungal plates were freeze-dried (8.45 g, dry weight) and extracted with MeOH (2 × 400 mL) for 4 h followed by CH_2_Cl_2_ (400 mL) overnight. Combined organic extracts were concentrated under reduced pressure to afford a brown oil (0.56 g). The crude product was subjected to C_8_ reversed-phase column chromatography eluted with a gradient of H_2_O/MeOH to afford five fractions (F1–F5). F4 was subjected to purification by Sephadex LH-20 and eluted with MeOH/5% CH_2_Cl_2_ to afford five fractions (A1–A5). Fractions A2–A4 were combined and triturated with CH_2_Cl_2_ to afford brefeldin A (**8**) (2.37 mg). F3 was subjected to purification by Sephadex LH-20, eluted with MeOH/5% CH_2_Cl_2_ to afford brefeldin C (**9**) (0.79 mg).

##### Brefeldin A (**8**)

[α]_D_^22^ = +58.2 (*c* = 0.22, MeOH) [lit [α]_D_^29^ = +92.2 (*c* = 0.51, MeOH) [7]]; ^1^H NMR (CD_3_OD, 400 MHz) δ 7.46 (1H, dd, *J* = 15.6, 3.1 Hz, H-3), 5.82 (1H, dd, *J* = 15.6, 2.2 Hz, H-2), 5.78–5.72 (1H, m, H-11), 5.27 (1H, dd, *J* = 15.3, 9.5 Hz, H-10) 4.82–4.78 (1H, m, H-15), 4.24–4.20 (1H, m, H-7), 4.05–4.02 (1H, m, H-4), 2.42–2.35 (1H, m, H-9), 2.16–2.10 (1H, m, H_2_-8a), 2.04–1.98 (2H, m, H_2_-6a, H_2_-12a), 1.89–1.80 (4H, m, H-5, H_2_-6b, H_2_-12b, H_2_-13a), 1.79–1.73 (1H, m, H_2_-14a), 1.62–1.55 (1H, m, H_2_-14b), 1.47–1.42 (1H, m, H_2_-8b), 1.24 (3H, d, *J* = 6.2 Hz, H_3_-16), 0.94–0.87 (1H, m, H_2_-13b); ^13^C NMR (CD_3_OD, 100 MHz) δ 167.3 (C-1), 154.1 (C-3), 136.9 (C-10), 130.4 (C-11), 116.6 (C-2), 75.8 (C-4), 72.2 (C-15), 72.0 (C-7), 52.3 (C-5), 44.4 (C-9), 43.3 (C-8), 40.7 (C-6), 34.0 (C-14), 32.4 (C-12), 27.1 (C-13), 20.1 (C-16); (+)-HRESIMS *m*/*z* 303.1569 [M + Na]^+^ (calcd for C_16_H_24_NaO_4_, 303.1567).

##### Brefeldin C (**9**)

[α]_D_^20^ = +83.1 (*c* = 0.22, MeOH) [lit [α]_D_^20^ = +121 (*c* = 0.07, MeOH) [11]]; ^1^H NMR (CD_3_OD, 400 MHz) δ 7.50 (1H, dd, *J* = 15.4, 3.3 Hz, H-3), 5.92–5.86 (1H, m, H-11), 5.87 (1H, dd, *J* = 15.4, 2.0 Hz, H-2), 5.26 (1H, dd, *J* = 15.1, 9.2 Hz, H-10), 4.82–4.80 (1H, m, H-4), 4.24–4.21 (1H, m, H-15), 2.82–2.78 (1H, m, H-9), 2.05–2.01 (3H, m, H_2_-6a, H_2_-8a, H_2_-12a), 1.94–1.86 (4H, m, H-5, H_2_-8b, H_2_-12b, H_2_-13a), 1.79–1.73 (2H, m, H_2_-7a, H_2_-14a), 1.65–1.57 (3H, m, H_2_-6b, H_2_-7b, H_2_-14b), 1.25 (3H, d, *J* = 6.4 Hz, H_3_-16), 0.99–0.97 (1H, m, H_2_-13b); (+)-HRESIMS *m*/*z* 287.1618 [M + Na]^+^ (calcd for C_16_H_24_NaO_3_, 287.1623).

#### 4.3.4. ICMP 20542—*Dactylonectria torresensis*

Nineteen PDA plates were inoculated with ICMP 20542 and incubated at room temperature for 5 weeks. Fully grown fungal plates were freeze-dried (15.26 g, dry weight) and extracted with MeOH (2 × 400 mL) for 4 h followed by CH_2_Cl_2_ (400 mL) overnight. Combined organic extracts were concentrated under reduced pressure to afford a brown oil (0.37 g). The crude product was subjected to C_8_ reversed-phase column chromatography eluted with a gradient of H_2_O/MeOH to afford five fractions (F1–F5). F4 was subjected to purification by Sephadex LH-20 and eluted with MeOH/5% CH_2_Cl_2_ to afford brefeldin A (**8**) (1.57 mg).

#### 4.3.5. ICMP 16794—*Ilyonectria europaea*

Ten PDA plates were inoculated with ICMP 16794 and incubated at room temperature for 4 weeks. Fully grown fungal plates were freeze-dried (5.42 g, dry weight) and extracted with MeOH (2 × 200 mL) for 4 h followed by CH_2_Cl_2_ (200 mL) overnight. Combined organic extracts were concentrated under reduced pressure to afford a brown oil (0.280 g). The crude product was subjected to purification by C_8_ reversed-phase column chromatography eluted with a gradient of H_2_O/MeOH to afford five fractions (F1–F5). Fraction F3 afforded radicicol (**10**) as a white solid (10.13 mg).

##### Radicicol (**10**)

[α]_D_^21^ = +96 (*c* = 0.25, CHCl_3_) [lit [α]_D_^20^ = +95.3 (*c* = 0.06, CHCl_3_) [8]; ^1^H NMR (CD_3_OD, 400 MHz) δ 7.59 (1H, dd, *J* = 16.1, 9.9 Hz, H-8), 6.46 (1H, s, H-15), 6.23 (1H, td, *J* = 10.7, 9.9 Hz, H-7), 6.09 (1H, d, *J* = 16.1 Hz, H-9), 5.77 (1H, dd, *J* = 10.7, 4.0 Hz, H-6), 5.38 (1H, m, H-2), 4.16 (1H, d, *J* = 16.2 Hz, H-11a), 3.92 (1H, d, *J* = 16.2 Hz, H-11b), 3.32 (1H, m, H-5), 3.05 (1H, dt, *J* = 8.6, 2.7 Hz, H-4), 2.41 (1H, dt, *J* = 14.8, 3.5 Hz, H-3a), 1.72 (1H, m, H-3b), 1.52 (3H, d, *J* = 6.6 Hz, H_3_-1); ^13^C NMR (CD_3_OD, 100 MHz) δ 200.6 (C-10), 169.7 (C-18), 160.9 (C-14, C-16), 140.6 (C-8), 136.6 (C-6), 135.2 (C-12), 131.4 (C-9), 130.8 (C-7), 116.9 (C-13), 108.4 (C-17), 104.5 (C-15), 71.8 (C-2), 56.9 (C-4), 56.5 (C-5), 46.8 (C-11), 37.5 (C-3), 18.7 (C-1); (+)-HRESIMS *m*/*z* 387.0614 [M + Na]^+^ (calcd for C_18_H_17_ClNaO_6_, 387.0606).

#### 4.3.6. ICMP 16795—*Ilyonectria liriodendri*

Four PDA plates were inoculated with ICMP 16795 and incubated at room temperature for 4 weeks. Fully grown fungal plates were freeze-dried (2.14 g, dry weight) and extracted with MeOH (2 × 200 mL) for 4 h followed by CH_2_Cl_2_ (200 mL) overnight. Combined organic extracts were concentrated under reduced pressure to afford a brown oil (0.189 g). The crude product was subjected to purification by Sephadex LH-20, eluted with MeOH, to afford four fractions (F1–F4). Purification of F2 by silica gel column chromatography, eluted with n-hexane/EtOAc (1:1), afforded a brown solid that was subsequently triturated with CH_2_Cl_2_ to afford radicicol (**10**) as a white solid (8.47 mg).

### 4.4. Antimicrobial Testing of Fungal Cultures

Fungal isolates were grown on PDA (Fort Richard, Auckland, New Zealand) prior to screening for antibacterial activity using a 24-well plate assay, using a modification of a protocol previously described [62]. Briefly, 0.5 mL aliquots of PDA agar were added to triplicate wells of a black 24-well plate (4titude, Millennium Science, Auckland, New Zealand) and allowed to set. With the aid of a sterile scalpel blade, fungal isolates grown on PDA were sectioned into cubes ≤5 mm in diameter and transferred to agar-filled wells of the 24-well plates, ensuring that each cube was placed fungus-side down and touching the agar. The inoculated 24-well screening plates were covered, sealed, and incubated at room temperature.

Fungal growth was monitored visually at regular intervals and recorded the time taken for them to either cover the entire well or to stop visibly growing. During this time, a 6 mm plug of agar was removed from each well using a biopsy punch twice. To screen for antimycobacterial activity, *M. abscessus* BSG301 [63] and *M. marinum* BSG101 [64] were resuspended in 0.8% Middlebrook 7H9 agar (Fort Richard, New Zealand) supplemented with 10% Middlebrook ADC enrichment media (Fort Richard, New Zealand) to a final concentration of approx. 10^7^ colony forming units (CFU)/mL for *M. abscessus* and 10^8^ CFU/mL for *M. marinum*. To screen for activity against *A. baumannii*, *E. coli*, and *P. aeruginosa*, bioluminescent derivatives of these bacteria were resuspended in 0.8% Mueller Hinton agar (Fort Richard, New Zealand) to achieve a final concentration of approx. 10^6^ colony forming units (CFU)/mL.

Thereafter, 50 μL of the bacterial–agar mixtures were pipetted into the cylindrical holes left after the removal of the fungal–agar plugs and allowed to set. The bacterial luminescence was measured at regular intervals using a Victor X-3 luminescence plate reader (PerkinElmer, Waltham, MA, USA) with an integration time of 1 s. Between measurements, plates were covered and incubated static at 28 °C for *M. marinum* and 37 °C for all of the other bacteria. Luminescence was also measured for bacteria inoculated into wells containing no fungus as the control.

### 4.5. Antimicrobial Assays of Pure Compounds

Antimicrobial evaluation of the pure compounds against *A. baumannii* ATCC 19606, *Candida albicans* ATCC 90028, *Cryptococcus neoformans* ATCC 208821, *E. coli* ATCC 25922, *K. pneumoniae* ATCC 700603, *P. aeruginosa* ATCC 27853, and *S. aureus* ATCC 43300 (MRSA) was undertaken at the Community for Open Antimicrobial Drug Discovery at The University of Queensland (St. Lucia, Queensland, Australia) according to standard protocols [65] as previously described [63,66,67].

Antimicrobial evaluation against *M. abscessus* and *M. marinum* was undertaken using in-house assays with the bioluminescent derivatives *M. abscessus* BSG301 [63] and *M. marinum* BSG101 [64]. Assays were performed as previously described [63,68]. Specifically, mycobacterial cultures were grown with shaking at 200 rpm in Middlebrook 7H9 broth (Fort Richard, Auckland, New Zealand) supplemented with 10% Middlebrook ADC enrichment media (Fort Richard, Auckland, New Zealand), 0.4% glycerol (Sigma-Aldrich, St. Louis, MO, USA), and 0.05% tyloxapol (Sigma-Aldrich, St. Louis, MO, USA). *M. abscessus* was grown at 37 °C and *M. marinum* at 28 °C. Cultures were grown until they reached the stationary phase (approximately 3–5 days for *M. abscessus* BSG301 and 7–10 days for *M. marinum* BSG101) and then diluted in MHB supplemented with 10% Middlebrook ADC enrichment media and 0.05% tyloxapol to give an optical density at 600 nm (OD_600_) of 0.001, which is the equivalent of ~10^6^ bacteria per mL. Pure compounds were dissolved in DMSO and added to the wells of a black 96-well plate (Nunc, Thermo Scientific, Waltham, MA, USA) at doubling dilutions with a maximum concentration of 128 mg/mL. Then, 50 mL of diluted bacterial culture was added to each well of the compound containing plates giving final compound concentrations of 0–64 mg/mL and a cell density of ~5 × 10^5^ CFU/mL. Rifampicin (Sigma-Aldrich, St. Louis, MO, USA) was used as the positive control at 1000 mg/mL for *M. abscessus* and 10 mg/mL for *M. marinum*. Between measurements, plates were covered, placed in a plastic box lined with damp paper towels, and incubated with shaking at 100 rpm at 37 °C for *M. abscessus* and 28 °C for *M. marinum*. Bacterial luminescence was measured at regular intervals over 72 h using a Victor X-3 luminescence plate reader with an integration time of 1 s. We defined the MIC as causing a 1-log reduction in light production, as previously described [69]. Experiments were carried out in triplicate and repeated if there was sufficient compound.

## 5. Conclusions

Investigation of several pathogens from the ICMP collection, *Alternaria radicina*, *Cercospora beticola*, *Dactylonectria macrodidyma*, *D. torresensis, Ilyonectria europaea,* and *I. liriodendra* afforded ten secondary metabolites, one of which was novel. Of the isolated metabolites, dehydro-curvularin (**6**) and radicicol (**10**) exhibited good activity against *M. marinum*; cercosporin (**7**) exhibited potent activity against MRSA; while brefeldin A (**8**) and radicicol (**10**) exhibited moderate antifungal activity against *C. albicans*. Although three of the compounds, **6–8**, were also found to be cytotoxic, **10** was non-cytotoxic and non-haemolytic, making it a promising candidate for further study.

## Figures and Tables

**Table 1:** Antimicrobial and antifungal activities of natural products **1–8** and **10**.

	MIC (µM)							HEK293 CC_50_ (µM) ^h^	HC_10_ (µM) ^i^
	S. *a* ^a^	E. *c* ^b^	K. *p* ^c^	P. *a* ^d^	A. *b* ^e^	C. *a* ^f^	C. *n* ^g^		
**1**	>184	>184	>184	>184	>184	>184	>184	n.t ^j^	n.t ^j^
**2**	>135	>135	>135	>135	>135	>135	>135	n.t ^j^	n.t ^j^
**3**	>79	>79	>79	>79	>79	>79	>79	n.t ^j^	n.t ^j^
**4**	>102	>102	>102	>102	>102	>102	>102	n.t ^j^	n.t ^j^
**5**	>109	>109	>109	>109	>109	>109	>109	n.t ^j^	n.t ^j^
**6**	>110	>110	>110	>110	>110	>110	>110	1.27	>110
**7**	**≤0.47**	>60	>60	>60	>60	>60	>60	25.33	>60
**8**	>114	>114	>114	>114	>114	**57**	>114	0.89	>114
**10**	>88	>88	>88	>88	>88	**44**	>88	>88	>88

Active compounds are shown in bold. All values are presented as the mean of two experiments. ^a^ *S. aureus* ATCC 43300 (MRSA) with vancomycin (MIC 0.7 μM) used as a positive control; ^b^ *E. coli* ATCC 25922 with colistin (MIC 0.1 μM); ^c^ *K. pneumoniae* ATCC 700603 with colistin (MIC 0.2 μM) as a positive control; ^d^ *P. aeruginosa* ATCC 27853 with colistin (MIC 0.2 μM); ^e^ *A. baumanii* ATCC 19606 with colistin (MIC 0.2 μM) as a positive control; ^f^ *C. albicans* ATCC 90028 with fluconazole (MIC 0.4 μM) as a positive control; ^g^ *C. neoformans* ATCC 208821 with fluconazole (MIC 26 μM) as a positive control; ^h^ Concentration of compound at 50% cytotoxicity on HEK293 human embryonic kidney cells with tamoxifen as the positive control (IC_50_ 24 μM); ^i^ Concentration of compound at 10% haemolytic activity on human red blood cells with melittin as the positive control (HC_10_ 0.95 μM); ^j^ Not tested.

## Data Availability

Raw data for the primary fungal screening and for the testing of pure compounds against *M. abscessus* and *M. marinum* are available on Figshare (https://doi.org/10.17608/k6.auckland.21714851, accessed on 20 January 2023).

## Supplementary Materials

The following supporting information can be downloaded at: https://www.mdpi.com/article/10.3390/molecules28031142/s1. Figure S1: ^1^H NMR spectrum (CD_3_OD, 400 MHz) of 1; Figure S2: ^13^C NMR spectrum (CD_3_OD, 100 MHz) of 1; Figure S3: COSY NMR spectrum (CD_3_OD) of 1; Figure S4: HSQC NMR spectrum (CD_3_OD) of 1; Figure S5: HMBC NMR spectrum (CD_3_OD) of 1; Figure S6. ^1^H NMR spectrum (CDCl_3_, 400 MHz) of 1.

## Sample Availability

Samples of compounds **4–7** and **10** are available from the authors.
