# Supplementary material for: Antimicrobial Natural Products from Plant Pathogenic Fungi
Source: Molecules. 2023 Jan 23;28(3):1142. doi: 10.3390/molecules28031142 (PMC9920077; doi:10.3390/molecules28031142)
Supplement: Supplementary file 1 [file molecules-28-01142-s001.zip › molecules-2128522-supplementary.pdf]

Supporting Information

Antimicrobial natural products from plant pathogenic fungi

Melissa M. Cadelis [1,2,*], Steven A. Li [1], Shara J. van de Pas [2], Alex Grey [2], Daniel Mulholland [2], Bevan S. Weir [3], Brent R. Copp [1,†] and Siouxsie Wiles [2,4*,†]

[1] School of Chemical Sciences, University of Auckland, Private Bag 92019, Auckland 1142, New Zealand
[2] Bioluminescent Superbugs Lab, School of Medical Sciences, University of Auckland, Private Bag 92019, Auckland 1142, New Zealand
[3] Manaaki Whenua-Landcare Research, Private Bag 92170, Auckland 1142, New Zealand
[4] Te Pūnaha Matatini Centre of Research Excellence in Complex Systems, New Zealand Corresponding

Author: Melissa M. Cadelis [1,2,*] and Siouxsie Wiles [2,*,†]
University of Auckland, Private Bag, 92019, Auckland 1142, New Zealand Email address: m.cadelis@auckland.ac.nz; s.wiles@auckland.ac.nz

Contents

Figure S1. ^{1}H NMR spectrum (CD_3OD, 400 MHz) of **1** **S2**
Figure S2. ^{13}C NMR spectrum (CD_3OD, 100 MHz) of **1** **S3**
Figure S3. COSY NMR spectrum (CD_3OD) of **1** **S4**
Figure S4. HSQC NMR spectrum (CD_3OD) of **1** **S5**
Figure S5. HMBC NMR spectrum (CD_3OD) of **1** **S6**
Figure S6. ^{1}H NMR spectrum ($CDCl_3$, 400 MHz) of **1** **S7**

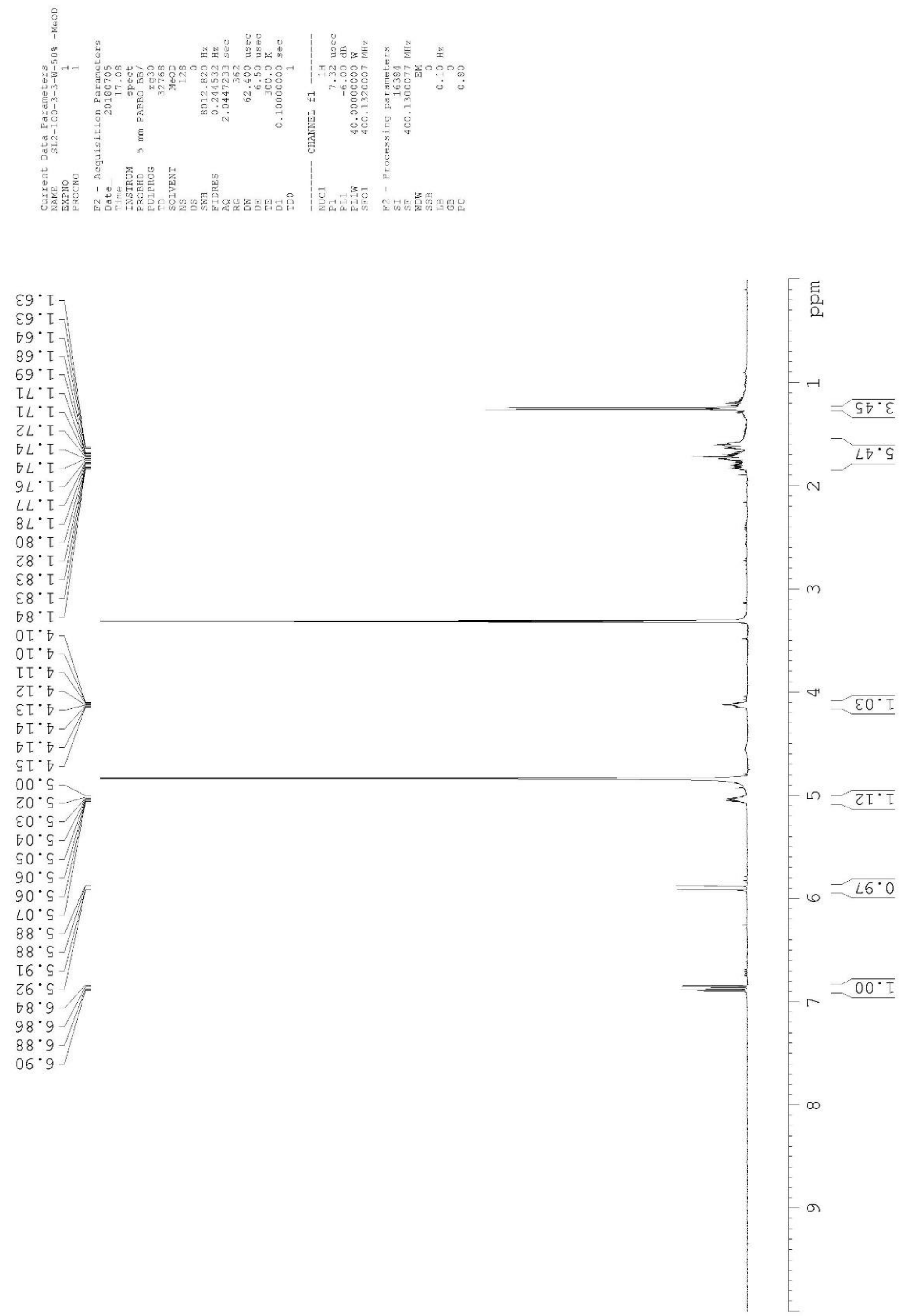

Figure S1. ^{1}H NMR spectrum (CD_3OD, 400 MHz) of **1**

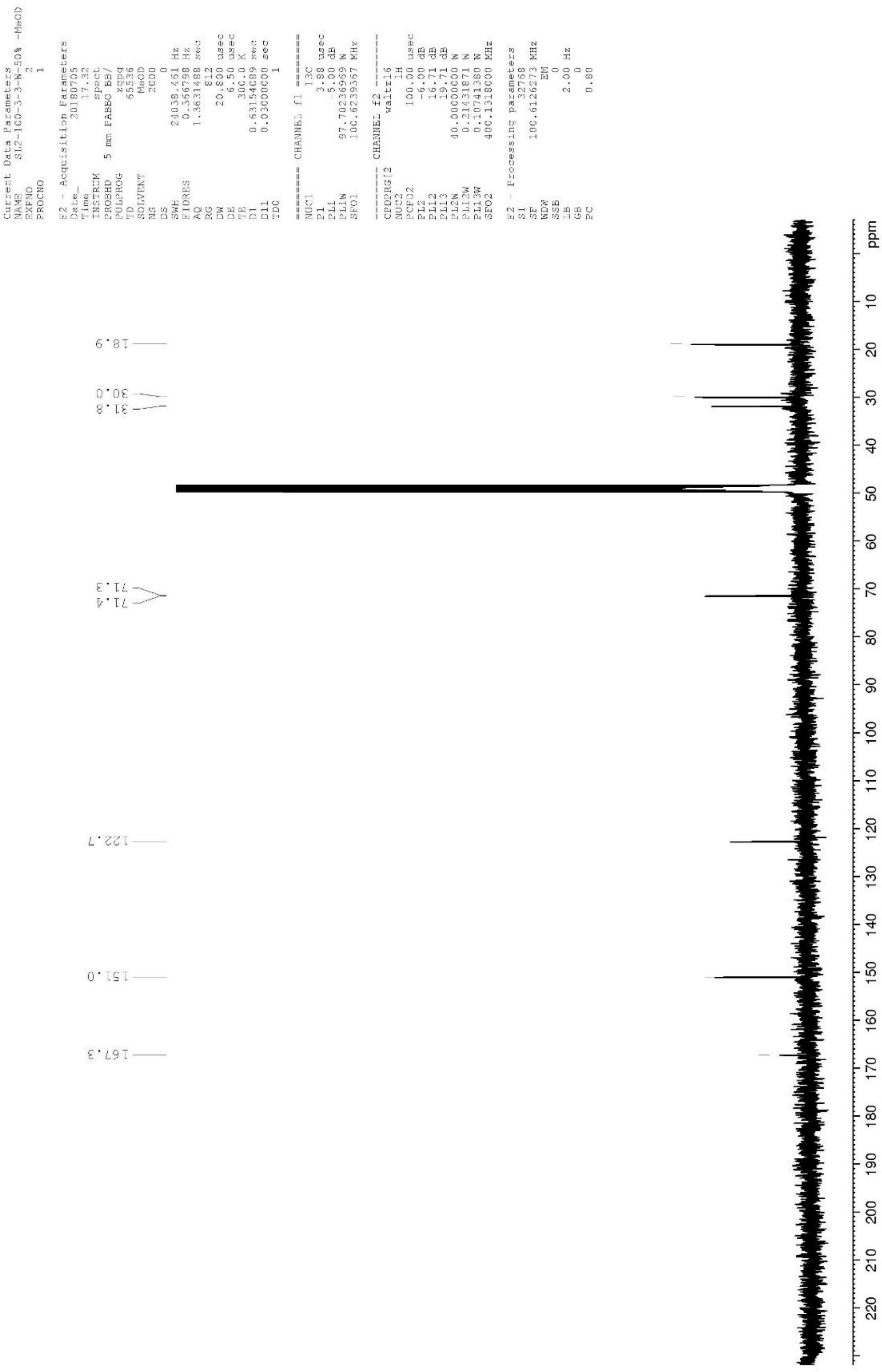

Figure S2. ^{13}C NMR spectrum (CD_3OD, 100 MHz) of **1**

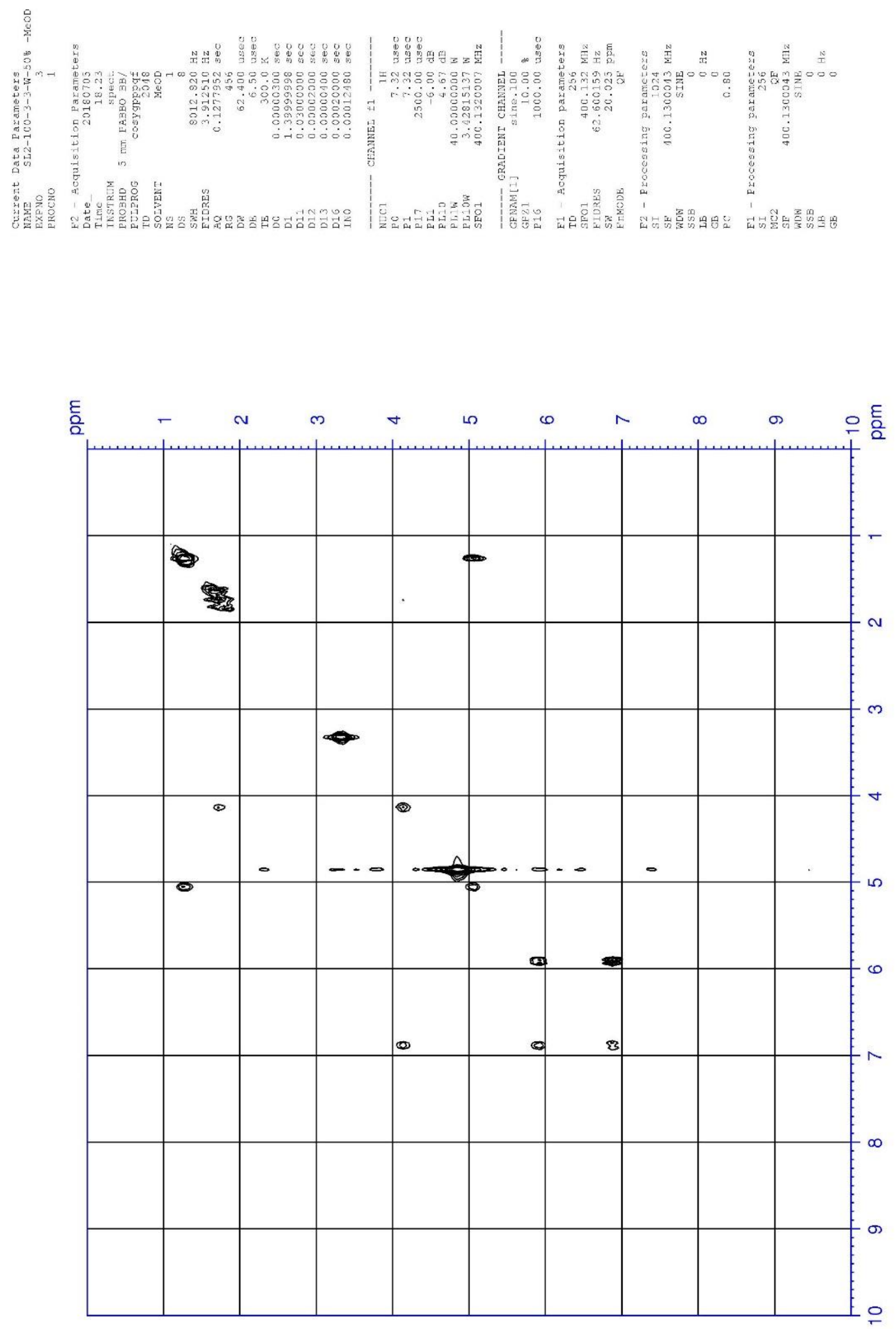

Figure S3. COSY NMR spectrum (CD_3OD) of **1**

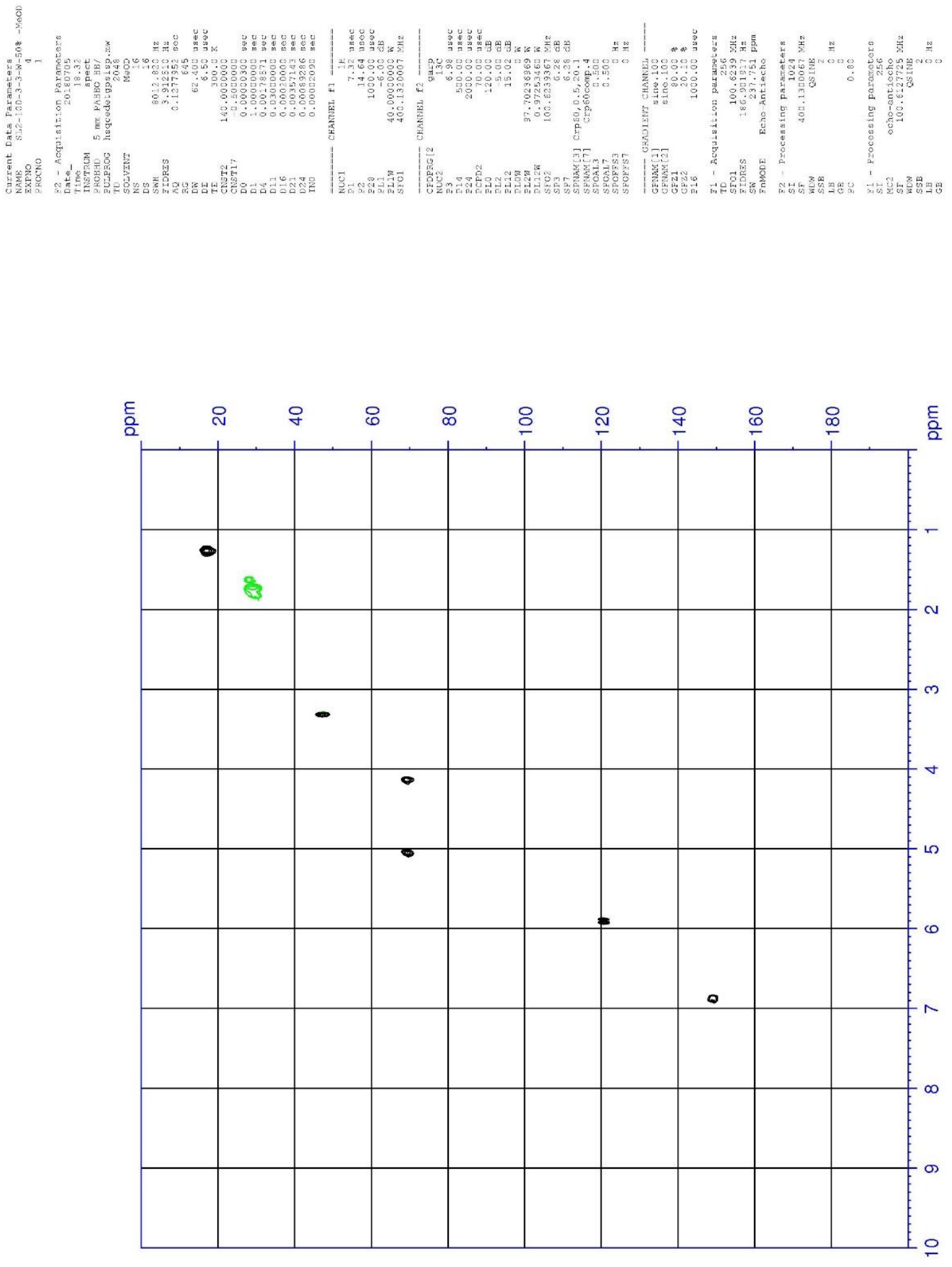

Figure S4. HSQC NMR spectrum (CD_3OD) of **1**

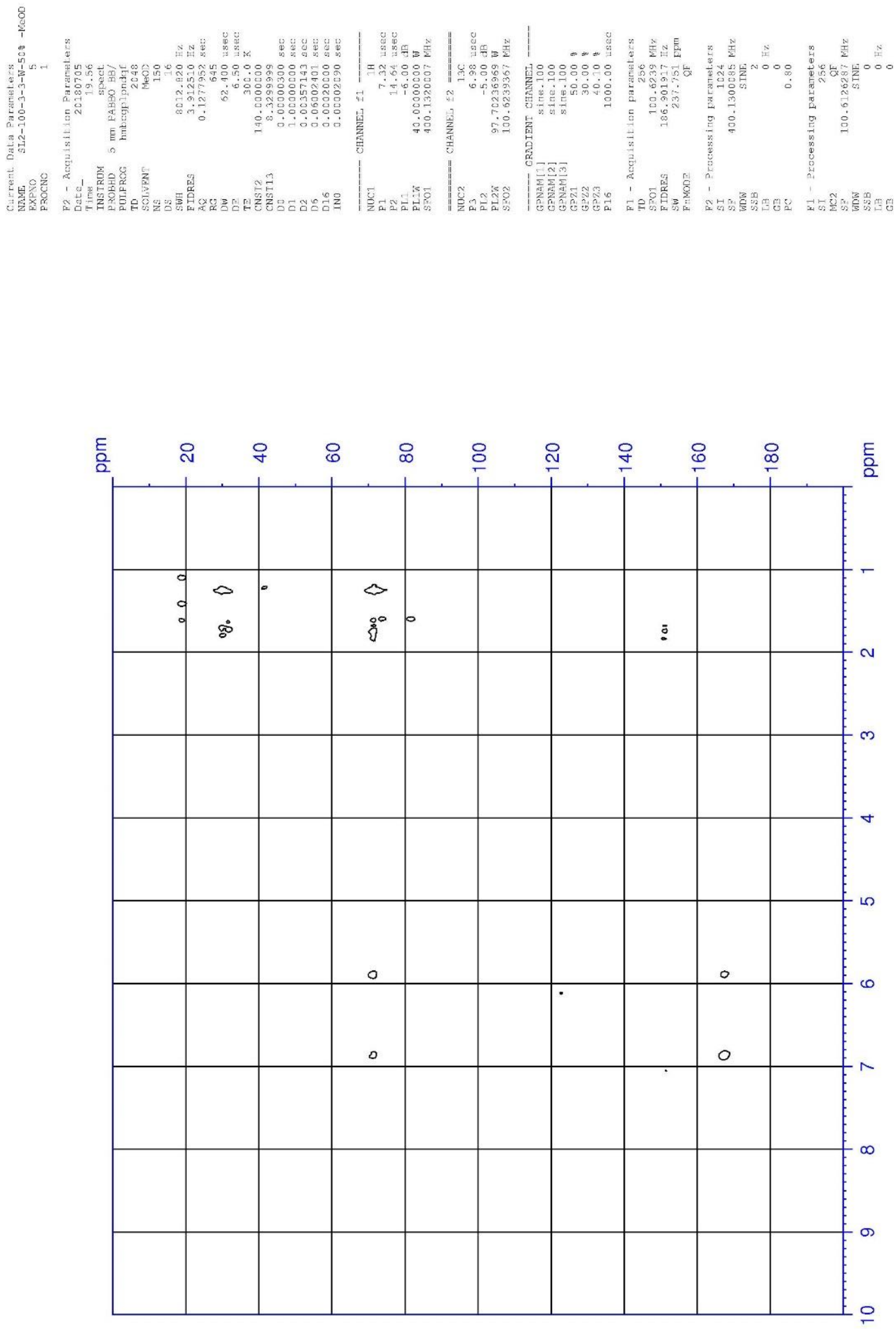

Figure S5. HMBC NMR spectrum (CD_3OD) of **1**

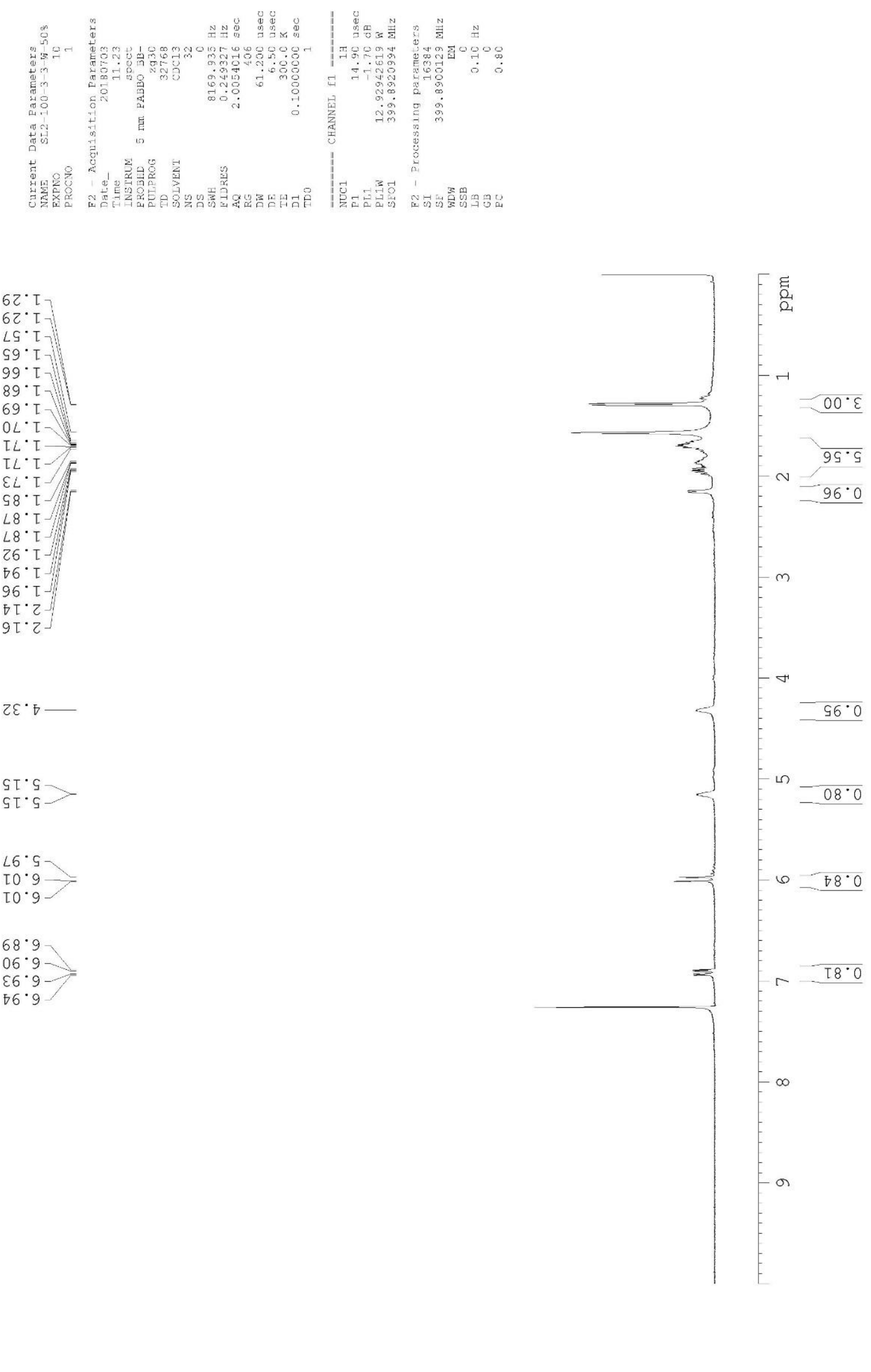

Figure S6. ^{1}H NMR spectrum ($CDCl_3$, 400 MHz) of **1**
